# Phylogenomics reveals extensive misidentification of fungal strains from the genus *Aspergillus*

**DOI:** 10.1128/spectrum.03980-23

**Published:** 2024-03-06

**Authors:** Jacob L. Steenwyk, Charu Balamurugan, Huzefa A. Raja, Carla Gonçalves, Ningxiao Li, Frank Martin, Judith Berman, Nicholas H. Oberlies, John G. Gibbons, Gustavo H. Goldman, David M. Geiser, Jos Houbraken, David S. Hibbett, Antonis Rokas

**Affiliations:** 1 Howards Hughes Medical Institute and the Department of Molecular and Cell Biology, University of California, Berkeley, California, USA; 2 Department of Biological Sciences, Vanderbilt University, Nashville, Tennessee, USA; 3 Evolutionary Studies Initiative, Vanderbilt University, Nashville, Tennessee, USA; 4 Department of Chemistry and Biochemistry, University of North Carolina at Greensboro, Greensboro, North Carolina, USA; 5 Department of Plant Pathology, University of California, Davis, California, USA; 6 USDA-ARS, Salinas, California, USA; 7 Shmunis School of Biomedicine and Cancer Research, George S. Wise Faculty of Life Sciences, Tel Aviv University, Ramat Aviv, Israel; 8 Department of Food Science, University of Massachusetts, Amherst, Massachusetts, USA; 9 Molecular and Cellular Biology Graduate Program, University of Massachusetts, Amherst, Massachusetts, USA; 10 Organismic and Evolutionary Biology Graduate Program, University of Massachusetts, Amherst, Massachusetts, USA; 11 Faculdade de Ciencias Farmacêuticas de Ribeirão Preto, Universidade de São Paulo, São Paulo, Brazil; 12 Department of Plant Pathology and Environmental Microbiology, Penn State University, University Park, Pennsylvania, USA; 13 Food and Indoor Mycology, Westerdijk Fungal Biodiversity Institute, Utrecht, The Netherlands; 14 Biology Department, Clark University, Worcester, Massachusetts, USA; 15 Heidelberg Institute for Theoretical Studies, Schloss-Wolfsbrunnenweg, Heidelberg, Germany; Institut Pasteur, Paris, France

**Keywords:** taxogenomics, pathogenicity, pathogen, virulence, plant pathogen, genomics, taxonomy, *Aspergillaceae*, *Penicillium*

## Abstract

Modern taxonomic classification is often based on phylogenetic analyses of a few molecular markers, although single-gene studies are still common. Here, we leverage genome-scale molecular phylogenetics (phylogenomics) of species and populations to reconstruct evolutionary relationships in a dense data set of 710 fungal genomes from the biomedically and technologically important genus *Aspergillus*. To do so, we generated a novel set of 1,362 high-quality molecular markers specific for *Aspergillus* and provided profile Hidden Markov Models for each, facilitating their use by others. Examining the resulting phylogeny helped resolve ongoing taxonomic controversies, identified new ones, and revealed extensive strain misidentification (7.59% of strains were previously misidentified), underscoring the importance of population-level sampling in species classification. These findings were corroborated using the current standard, taxonomically informative loci. These findings suggest that phylogenomics of species and populations can facilitate accurate taxonomic classifications and reconstructions of the Tree of Life.

**IMPORTANCE:**

Identification of fungal species relies on the use of molecular markers. Advances in genomic technologies have made it possible to sequence the genome of any fungal strain, making it possible to use genomic data for the accurate assignment of strains to fungal species (and for the discovery of new ones). We examined the usefulness and current limitations of genomic data using a large data set of 710 publicly available genomes from multiple strains and species of the biomedically, agriculturally, and industrially important genus *Aspergillus*. Our evolutionary genomic analyses revealed that nearly 8% of publicly available *Aspergillus* genomes are misidentified. Our work highlights the usefulness of genomic data for fungal systematic biology and suggests that systematic genome sequencing of multiple strains, including reference strains (e.g., type strains), of fungal species will be required to reduce misidentification errors in public databases.

## INTRODUCTION

Species determination is the foundation for evolutionary studies (1–4). Over the years, several different species concepts have been proposed (1). Due to the microscopic nature of many fungi and the lack of known sexual cycles for some species (5), species delimitation in the Kingdom Fungi has relied, in addition to cultural growth and micromorphological data, on molecular phylogenetics and the adoption of universal molecular barcodes (6) such as the ribosomal large and small subunit; the nuclear ribosomal internal transcribed spacer regions including the 5.8 S rDNA (ITS); the RNA polymerase II large subunit and core subunits; and minichromosome maintenance complex component 7 (7, 8).

Typically, the sequences of one or more barcode loci are obtained from a strain of interest. Next, orthologous sequences are inferred based on sequence similarity from databases such as the National Center for Biotechnology Information (NCBI). Software is subsequently used to infer phylogenetic trees for each sequence, representing a hypothesis of the evolutionary history of these species (8). Despite the potential for genome-scale data to facilitate fungal taxonomy, current practices typically do not rely on whole-genome data, in part because of the sparsity of available genome sequences across the fungal tree of life (3, 4, 9–17).

Species in the genus *Aspergillus* are of medical, agricultural, and biotechnological significance. *Aspergillus fumigatus* and *Aspergillus flavus* are pathogens and allergens, and produce mycotoxins (18, 19). *Aspergillus niger* is an industrial workhorse, and *Aspergillus oryzae* is used to produce fermented foods like soy sauce and sake (20, 21). Accurate identification of *Aspergillus* fungi with barcode loci is often challenging (13, 15–17, 22, 23) but, importantly, because even closely related species can differ in drug resistance profiles and ability to cause disease (24–26). For example, clinical strains of *Aspergillus nomius* and *Aspergillus tamarii* have been misidentified as *A. flavus* (27). In the clinic, inaccurate species determination could lead to misguided disease management strategies due to differences in intrinsic drug resistance levels between species (23). For example, *Aspergillus latus*, commonly misidentified as *Aspergillus nidulans*, is more resistant to the antifungal drug caspofungin than *A. nidulans* (28). However, there is no current consensus for the levels of barcode sequence divergence required to consider two distinct fungal clades as different species; for example, calmodulin gene sequences of *Aspergillus labruscus* and *Aspergillus oerlinghausenensis—*two recently described species of *Aspergillus—*share 85% and 97.3% sequence similarity to their closest relatives, *Aspergillus homomorphus* and *A. fumigatus,* respectively (29, 30).

Reconstructing deeper evolutionary relationships from a few molecular markers can also be challenging. Divergences among sections—a secondary taxonomic rank above the species and below the genus ranks—have been debated. For example, the sections *Nigri, Ochraceorosei, Flavi, Circumdati, Candidi,* and *Terrei* were inferred to be monophyletic based on analyses of four loci from 81 taxa (31, 32) but topology tests using a 1,668-gene matrix from a different 81-taxon data set rejected the monophyly of these lineages (33). Accurate reconstructions of the *Aspergillus* phylogeny will facilitate our understanding of how biomedically and technologically relevant traits, such as antimicrobial resistance, evolved.

Here, we present a dense phylogenomic tree inferred from a 1,362-gene matrix from 710 *Aspergillus* genome sequences spanning 98 species and population data for 36 species, more than doubling the number of species analyzed in previous genome-scale studies (33–35) and capturing roughly one-quarter of all known species in the genus (36, 37). The new phylogeny reveals that phylogenomics using species and populations can facilitate strain classification and resolve taxonomic controversies while identifying new ones. Moreover, phylogenomic analyses revealed 7.59% of strains (55/725) were previously misidentified. These findings were further corroborated using taxonomically informative loci, the current gold standard in the field of systematics.

## RESULTS AND DISCUSSION

### A phylogenomic tree of *Aspergillus*

**Fig 1:**
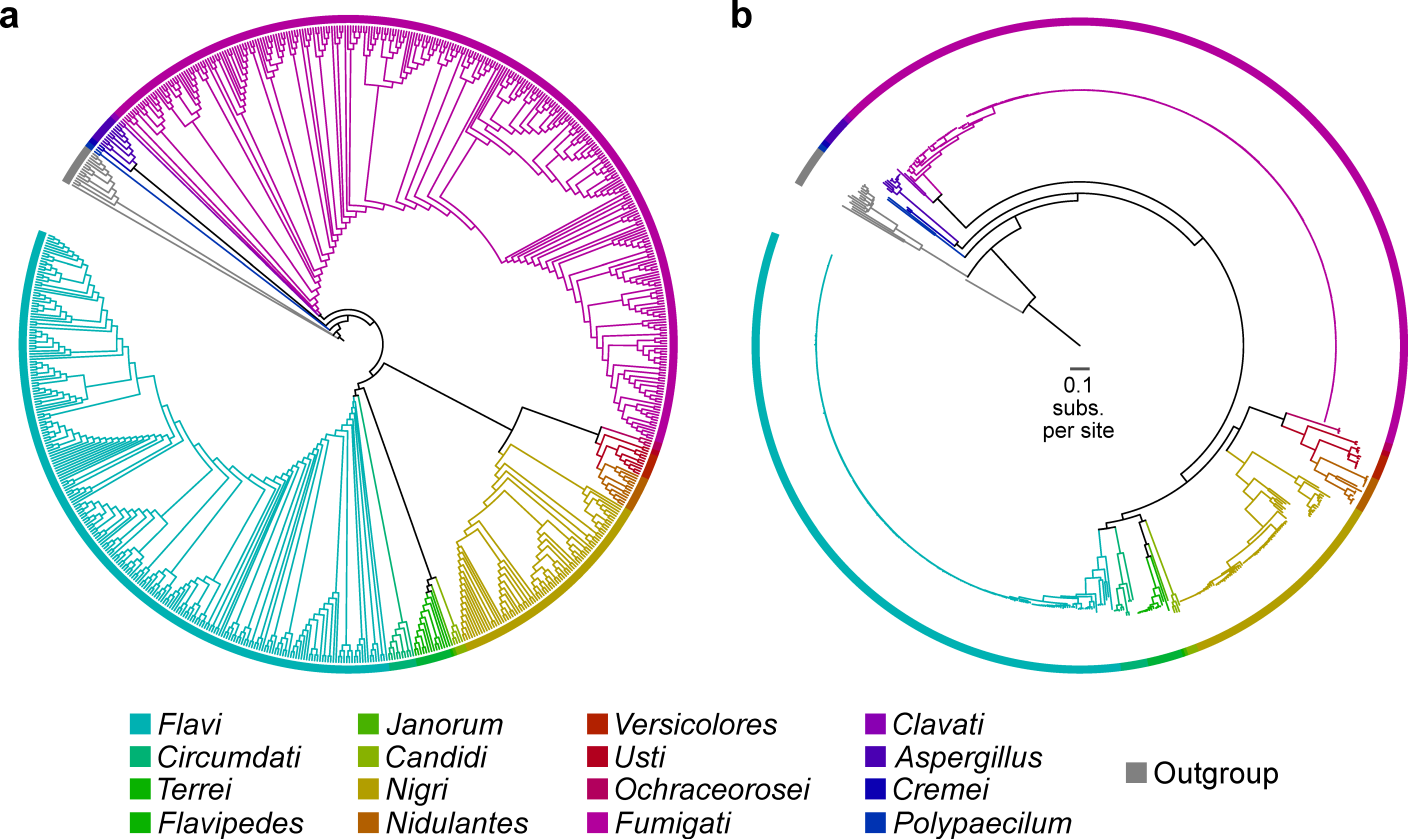
Phylogenomic tree of 725 genomes based on analysis of 1,362 genes (6,378,237 nucleotide sites). The evolutionary history of 710 *Aspergillus* species and 15 outgroup genomes was reconstructed from a 1,362-gene matrix. The phylogeny is depicted without branch lengths (**a**) and with branch lengths, representing substitutions per site (**b**). Colors represent different sections—taxonomic ranks above species and below genus. Note, *Versicolores* is a series within the section *Nidulantes*.

**Fig 2:**
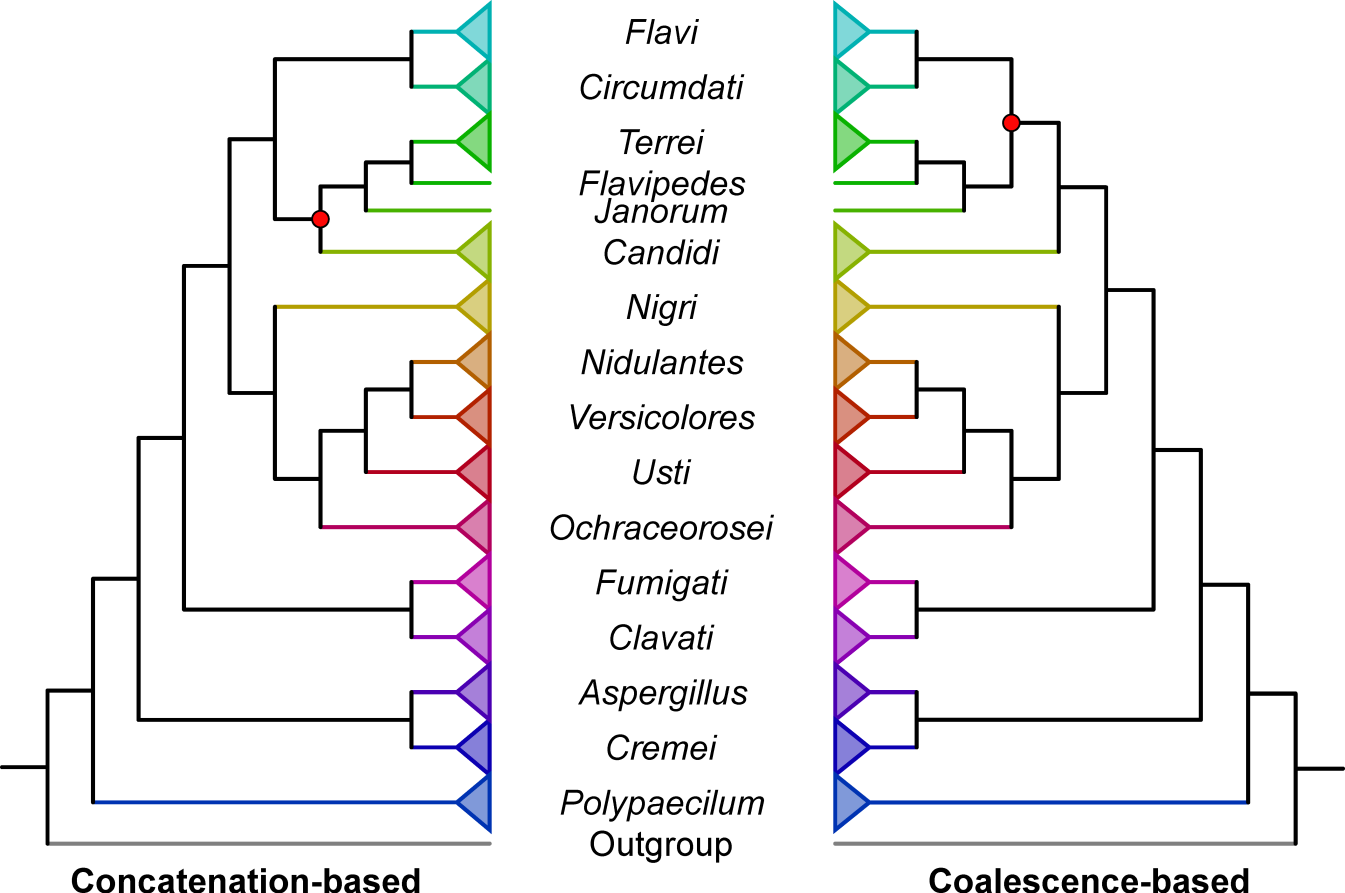
Concatenation- (left) and coalescence-based (right) phylogenies of taxonomic sections in the genus *Aspergillus* are highly congruent. The evolutionary relationships among sections are depicted. Species-level concatenation- and coalescence-based phylogenies differed at two bipartitions (represented by a red dot). Branch lengths and triangle sizes have no meaning. Note, *Versicolores* is a series within the section *Nidulantes*.

The evolutionary history of 725 genomes (710 *Aspergillus* genomes; 15 outgroup genomes) was reconstructed using maximum likelihood analysis of a 1,362-gene data matrix with 6,378,237 nucleotide sites (Fig. 1; Fig. S1 at https://doi.org/10.6084/m9.figshare.21382131). The 725 genomes represent public whole genome assemblies available through the NCBI (https://www.ncbi.nlm.nih.gov/) that also passed quality-control measures (see Materials and Methods; Tables S1 and S2 at https://doi.org/10.6084/m9.figshare.21382131). Based on the NCBI-provided taxonomic information, the data set includes 115 *Aspergillus* and 14 *Penicillium* species. Note that analysis presented later in our manuscript identified one *Aspergillus* genome (strain MCCF 102) as *Paecilomyces formosus*; thus, the total number of outgroup genomes is 15. The genomes of two or more strains were available for 36 *Aspergillus* species, but the depth of strain sampling varied (Table S4 at https://doi.org/10.6084/m9.figshare.21382131). Sampling was densest for *A. fumigatus* (*N* = 275), *A. flavus* (*N* = 105), *A. oryzae* (*N* = 97), and *A. niger* (*N* = 24). Sixteen species had genome sequences from two representative strains available. Twelve strains were of unknown species but were reportedly from the genus *Aspergillus*. The data set spanned 17 *Aspergillus* sections. Higher-order relationships among sections were examined using concatenation- and coalescence-based tree inference approaches (38, 39). The phylogenies inferred with both approaches were highly congruent, differing by only one bipartition (Fig. 2).

### Phylogenomics sheds new light on *Aspergillus* strain identification

Phylogenomic analyses reveal that half of the 36 species with multiple strains were not monophyletic (Table S5 at https://doi.org/10.6084/m9.figshare.21382131). Although monophyly is not required for species delimitation under certain species concepts (40, 41), this observation was surprising for *Aspergillus* because new species descriptions typically include molecular phylogenetic evidence, and suggests that cryptic speciation and/or species misidentification may be rampant. Cross-referencing these findings with strain determination via taxonomically informative loci indicated that 55 strains (55/725 or 7.59%) had been misidentified (Table 1). The high percentage of misidentified strains underscores both the magnitude of the problem as well as the importance of population sampling for accurate strain identification. Here, we highlight eight cases that illustrate this issue.

**TABLE 1:** Summary of 55 misidentified isolates^*a*^

Updated genus and species	Strain identifier	Updated section	NCBI accession	Genus and species on NCBI	Section based on NCBI data
*Aspergillus pseudoglaucus*	CBS 112.26	*Aspergillus*	GCA_020284055.1	*Aspergillus brunneus*	*Aspergillus*
*Aspergillus neotritici*	UdeA_Ac1	*Candidi*	GCA_009812425.1	*Aspergillus tritici*	*Candidi*
*Aspergillus pallidofulvus*	AO.MF010	*Circumdati*	GCA_005784425.1	*Aspergillus ochraceus*	*Circumdati*
*Aspergillus westerdijkiae*	fc-1	*Circumdati*	GCA_004849945.1	*Aspergillus ochraceus*	*Circumdati*
*Aspergillus sclerotiorum*	NIBRFGC000004109	*Circumdati*	GCA_002215965.1	*Aspergillus persii*	*Circumdati*
*Aspergillus subramanianii*	HBR18	*Circumdati*	GCA_000530345.1	*Aspergillus sclerotiorum*	*Circumdati*
*Aspergillus europaeus*	IBT 35662	*Cremei*	GCA_019976455.1	*Aspergillus* sp. subgen. *Cremei*	*Cremei*
*Paecilomyces formosus*	MCCF 102	Not applicable	GCA_018167545.1	*Aspergillus* sp.	Unknown
*Aspergillus salisburgensis*	HF37	*Polypaecilum*	GCA_003698115.1	*Aspergillus* sp.	Unknown
*Aspergillus luteovirescens*	NRRL 26010	*Flavi*	GCF_001792695.1	*Aspergillus bombycis*	*Flavi*
*Aspergillus alliaceus*	FRR 5400	*Flavi*	GCA_013421405.2	*Aspergillus burnettii*	*Flavi*
*Aspergillus minisclerotigenes*	E1293	*Flavi*	GCA_013145855.1	*Aspergillus flavus*	*Flavi*
*Aspergillus minisclerotigenes*	E1406	*Flavi*	GCA_013146155.1	*Aspergillus flavus*	*Flavi*
*Aspergillus minisclerotigenes*	E1316	*Flavi*	GCA_013145865.1	*Aspergillus flavus*	*Flavi*
*Aspergillus minisclerotigenes*	E1288	*Flavi*	GCA_011420435.1	*Aspergillus flavus*	*Flavi*
*Aspergillus minisclerotigenes*	E1376	*Flavi*	GCA_013146015.1	*Aspergillus flavus*	*Flavi*
*Aspergillus pseudonomiae*	HBR9	*Flavi*	GCA_000531055.1	*Aspergillus nomiae*	*Flavi*
*Aspergillus flavus* or *Aspergillus oryzae*	NRRL 2999	*Flavi*	GCA_012897115.1	*Aspergillus parasiticus*	*Flavi*
*Aspergillus flavus* or *Aspergillus oryzae*	E1365	*Flavi*	GCA_013145995.1	*Aspergillus parasiticus*	*Flavi*
*Aspergillus flavus* or *Aspergillus oryzae*	GbtcF2	*Flavi*	GCA_019176375.1	*Aspergillus* sp.	*Unknown*
*Aspergillus flavus* or *Aspergillus oryzae*	ATCC 12892	*Flavi*	GCA_002894705.1	*Aspergillus* sp.	*Unknown*
*Aspergillus parasiticus* or *Aspergillus sojae* (should be *A. transmontanensis*; strain contaminated)	CBS 130015	*Flavi*	GCA_009193505.1	*Aspergillus transmontanensis*	*Flavi*
*Aspergillus fumigatus*	NRRL 5109	*Fumigati*	GCA_003116565.1	*Aspergillus neoellipticus*	*Fumigati*
*Aspergillus fumigatus*	GbtcF1	*Fumigati*	GCA_019176365.1	*Aspergillus* sp.	Unknown
*Aspergillus unguis*	JS3-R5	*Nidulantes*	GCA_019775315.1	*Aspergillus* sp.	Unknown
*Aspergillus luchuensis*	JCM 22320	*Nigri*	GCA_001599415.1	*Aspergillus awamori*	*Nigri*
*Aspergillus niger*	IFM 58123	*Nigri*	GCA_003850985.1	*Aspergillus awamori*	*Nigri*
*Aspergillus tubingensis*	CBS 115574	*Nigri*	GCF_003184835.1	*Aspergillus costaricaensis*	*Nigri*
*Aspergillus brunneoviolaceus*	CBS 313.89	*Nigri*	GCF_003184825.1	*Aspergillus fijiensis*	*Nigri*
*Aspergillus tubingensis*	CBS 115656	*Nigri*	GCF_003184625.1	*Aspergillus neoniger*	*Nigri*
*Aspergillus luchuensis*	RAF106	*Nigri*	GCA_011316255.1	*Aspergillus niger*	*Nigri*
*Aspergillus tubingensis*	3.316	*Nigri*	GCA_013618955.1	*Aspergillus niger*	*Nigri*
*Aspergillus tubingensis*	An76	*Nigri*	GCA_001515345.1	*Aspergillus niger*	*Nigri*
*Aspergillus niger*	ATCC 13157	*Nigri*	GCA_003344505.1	*Aspergillus phoenicis*	*Usti*
*Aspergillus luchuensis*	CBS 112811	*Nigri*	GCF_003184755.1	*Aspergillus piperis*	*Nigri*
*Aspergillus japonicus*	CBS 115571	*Nigri*	GCA_003184705.1	*Aspergillus violaceofuscus*	*Nigri*
*Aspergillus niger*	CCMB 674	*Nigri*	GCA_009761105.1	*Aspergillus welwitschiae*	*Nigri*
*Aspergillus niger*	IHEM 2864	*Nigri*	GCA_012275225.1	*Aspergillus welwitschiae*	*Nigri*
*Aspergillus niger*	CBS 139.54b	*Nigri*	GCF_003344945.1	*Aspergillus welwitschiae*	*Nigri*
*Aspergillus niger*	ITEM 11945	*Nigri*	GCA_012365075.1	*Aspergillus welwitschiae*	*Nigri*
*Aspergillus ochraceoroseus*	SRRC1468	*Ochraceorosei*	GCA_000986645.1	*Aspergillus rambellii*	*Ochraceorosei*
*Aspergillus floccosus*	IMV 01167	*Terrei*	GCA_001931935.1	*Aspergillus* aff. *floccosus*	Unknown
*Aspergillus* sp*.* nov.	MEXU 27854	*Terrei*	GCA_019721355.1	*Aspergillus* sp.	Unknown
*Aspergillus terreus*	A31	*Terrei*	GCA_016162245.1	*Aspergillus* sp.	Unknown
*Aspergillus pseudoterreus*	IFO 6365	*Terrei*	GCA_009932835.1	*Aspergillus terreus*	*Terrei*
*Aspergillus pseudoterreus*	TN-484	*Terrei*	GCA_009014675.2	*Aspergillus terreus*	*Terrei*
*Aspergillus pseudodeflectus*	ADI1	*Usti*	GCA_020615375.1	*Aspergillus* sp.	Unknown
*Aspergillus versicolor*	UdeA_Aid1	*Nidulantes*	GCA_009812435.1	*Aspergillus amoenus*	*Versicolores*
*Aspergillus creber*	MK2	*Nidulantes*	GCF_016861865.1	*Aspergillus puulaauensis*	*Versicolores*
*Aspergillus creber*	MA 6037	*Nidulantes*	GCA_003138035.1	*Aspergillus* sp.	*Versicolores*
*Aspergillus creber*	2663	*Nidulantes*	GCA_016880755.1	*Aspergillus* sp.	*Versicolores*
*Aspergillus sydowii*	ATCC 9577	*Nidulantes*	GCA_020284045.1	*Aspergillus versicolor*	*Versicolores*
*Penicillium rubens*	P2niaD18	*Chrysogena*	GCA_000710275.1	*Penicillium chrysogenum*	*Chrysogena*
*Penicillium brevistipitatum*	IBT 31321	*Robsamsonia*	GCA_002072405.1	*Penicillium coprophilum*	*Robsamsonia*

^*a*^ The first column refers to the updated genus and species name. The second column is the strain identifier. The third column is the updated section. The fourth column is the NCBI accession for the genome assembly. The fifth and sixth columns are the original genus, species, and corresponding section designation. Note, *Versicolores* is a series within the section *Nidulantes*.

#### *Aspergillus fumigatus* and *Aspergillus neoellipticus*

The phylogenetic placement of *A. neoellipticus* has been debated and bears on disease management strategies. Some studies suggest that *A. neoellipticus* is distinct from the major human pathogen *A. fumigatus*, based on the analysis of five loci (42). Other studies suggested that *A. neoellipticus* is conspecific to *A. fumigatus* based on partial single-gene sequences and random amplified polymorphic DNA (42–45). Resolving if *A. neoellipticus* is a separate species bears on our understanding of the evolution of pathogenicity in *Aspergillus*. Specifically, if *A. neoellipticus* can cause disease, as implicated in the ex-type strain NRRL 5109 (45), which was isolated from a case of chronic emphysema, the ability to cause disease would have evolved in the ancestor between *A. fumigatus* and *A. neoellipticus*; if not, pathogenicity would have only evolved in *A. fumigatus*.

**Fig 3:**
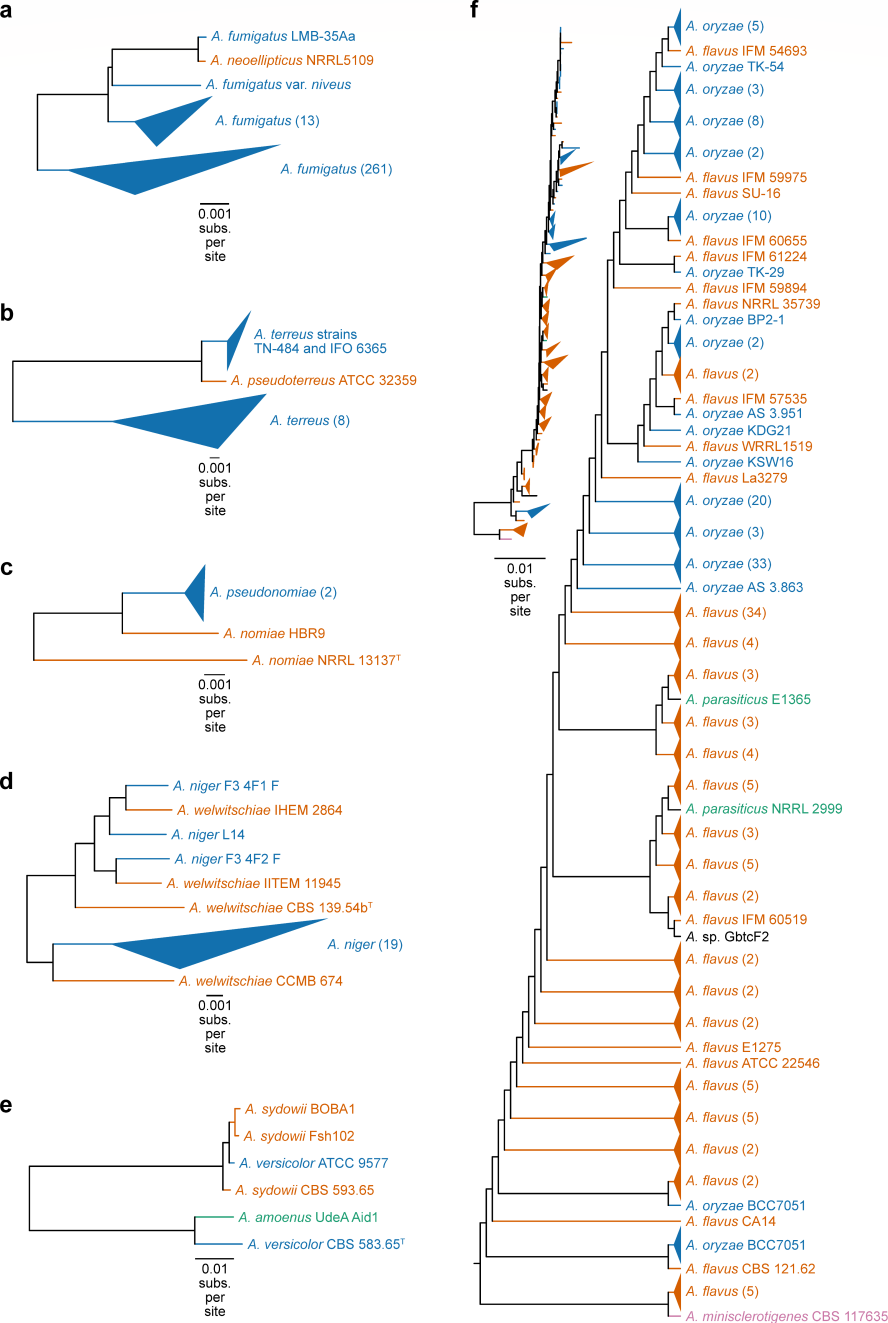
Phylogenomics underscores known taxonomic uncertainties and reveals new ones. (**a**) *A. neoellipticus* NRRL 5109 is an *A. fumigatus* strain or the clade formed by the 16 *A*. *fumigatus* strains and *A. neoellipticus* NRRL 5109 is, in fact, *A. neoellipticus*, a species sister to *A. fumigatus*. (**b**) Strain misidentification occurs between *Aspergillus pseudoterreus* and *Aspergillus terreus* and (**c**) *Aspergillus pseudonomiae* and *Aspergillus nomiae*. (**d**) Strains identified as *Aspergillus welwitschiae* are *A. niger*. (**e**) *Aspergillus* strain ATCC 9577 is misidentified as *Aspergillus versicolor* but is *Aspergillus sydowii*. (**f**) Strains of *A. oryzae*, *A. flavus*, *Aspergillus parasiticus*, and *Aspergillus minisclerotigenes* appear to have polyphyletic origins, a result that is likely due to extensive strain misidentification [e.g., see also Houbraken et al. (22), detailing misidentification of five strains of *A. minisclerotigenes* as *A. flavus* (22)]. Topologies presented were inferred using the concatenation approach. See Fig. S3 at https://doi.org/10.6084/m9.figshare.21382131 for topologies inferred using coalescence. Different colors represent different species. Isolates with no known species are depicted in black. Triangles represent collapsed linages with multiple isolates. The number of isolates in each collapsed lineage is shown next to the species name in parentheses. See Table 1 for the revised taxonomy.

Phylogenomic analyses of species in section *Fumigati* (including four strains of *A. fumigatus* and one strain of *A. neoellipticus*) failed to resolve this ongoing debate because *A. neoellipticus* was inferred as a sister to *A. fumigatus* (46). The combined use of genome-scale data and extensive population-level sampling of 275 *A*. *fumigatus* strains in the analyses found that *A. neoellipticus* NRRL 5109 is nested within a clade of 16 isolates of *A. fumigatus* (Fig. 3a); the remaining 261 *A*. *fumigatus* isolates form the sister lineage. These results can be interpreted in two ways. Either *A. neoellipticus* NRRL 5109 is an isolate of *A. fumigatus*, or the clade formed by the 16 *A*. *fumigatus* strains and *A. neoellipticus* NRRL5109 is, in fact, *A. neoellipticus*, a species sister to *A. fumigatus*.

Resolving this taxonomic controversy may require additional taxonomically informative features. These features may include secondary metabolite production, minimum inhibit concentration profiles, and morphological features (e.g., spore size and shape). Moreover, examinations of genome sequencing among additional strains coupled with concordance analysis of gene histories among representative *A. fumigatus*/*A. neoellipticus* strains and other closely related species, such as *A. oerlinghausenensis* and *Aspergillus fischeri* (25) may help elucidate species boundaries. Moreover, biological species concepts, examined through mating compatibility tests (47), may also prove helpful.

#### *Aspergillus pseudoterreus* and *Aspergillus pseudonomiae*

A similar situation, but with much lower levels of sampling, exists for two other pairs of species: *Aspergillus terreus* and *A. pseudoterreus* (Fig. 3b) and *A. nomiae* and *A. pseudonomiae* (Fig. 3c). For *A. terreus* strains TN-484 and IFO 6365, our data corroborate recent findings that these are misidentified and should be *A. pseudoterreus*, suggesting misidentified information has persisted in databases (22). For *A. pseudonomiae*, phylogenomics revealed strain HBR9—which was initially labeled as *A. nomiae—*is monophyletic with *A. pseudonomiae* isolates suggesting the strain is mislabeled (Fig. 3c). Sequence similarity searches of the taxonomically informative locus, β-tubulin, also suggest HBR9 is *A. pseudonomiae* (Table S3 at https://doi.org/10.6084/m9.figshare.21382131).

#### Aspergillus niger

Species from section *Nigri* are similar both in their morphology and in their molecular barcode sequences (48). Although additional barcode sequences, such as beta-tubulin and calmodulin gene sequences help differentiate species within the section, the high similarity of certain taxonomically informative loci presents an opportunity to demonstrate the power and utility of genome-scale data. As an example, we highlight strain misidentification between *A. niger* and *Aspergillus welwitschiae*.

*A. niger*, a species from the section *Nigri* (49), has been considered a prominent pathogen of sisal (*Agave sisalana*), an industrial crop used in the textile industry (50). However, recent molecular phylogenetic analysis suggests that the main etiological agent of sisal bole rot disease is *A. welwitschiae,* not *A. niger* (50). Examination of the evolutionary history of *A. niger* and *A. welwitschiae,* using phylogenomic analyses that sampled multiple strains from both species, identified four cases of strain misidentification, concerning strains CCMB 674, IHEM 2864, CBS 139.54b, and ITEM 11945 (Fig. 3d). Sequencing similarity searches of taxonomically informative loci further corroborated this finding (Table S3 at https://doi.org/10.6084/m9.figshare.21382131). Although some reports have recently suggested to unite *A. niger* and *A. welwitschiae* into a single species (51), this proposal has yet to be widely adopted. Our phylogenomic analyses support the presence of two distinct clades; however, a broader strain sampling covering a higher diversity should be conducted to gain better insight. An alternative interpretation is that *A. niger* may have two distinct populations (Fig. 3d). Either way, results from phylogenomic analyses highlight how genome-wide analyses inform our understanding of intra-species genetic diversity and species boundaries. Until the taxonomy is resolved, uncertainty confounds studies of sisal bole rot, which could have economic ramifications.

#### Aspergillus sydowii

Another instance of putative misidentification concerns *Aspergillus versicolor* ATCC 9577, which is conspecific with *A. sydowii* strains, suggesting that strain ATCC 9577 is, in fact, *A. sydowii* (Fig. 3e). This observation was further supported using sequence similarity searches of taxonomically informative loci (Table S3 at https://doi.org/10.6084/m9.figshare.21382131). ATCC 9577 and CBS 583.65 are derived from the same sample material and serve as ex-neotype strains of *A. versicolor* (52); the availability of two different genome sequences suggests contamination or erroneous metadata for the former strain. The close relationship of CBS 583.65^T^ with *Aspergillus amoenus* is supported by previous taxonomic studies (52, 53).

These findings suggest that the entire clade may benefit from taxonomic revision and closer scrutiny of strain identity. To this point, a recent analysis of a five-gene, 213-taxon data set proposed species in the series *Versicolores* (section *Nidulantes*) be reduced from 17 species to four, citing intraspecific variation as a driver for over-splitting species boundaries by taxonomists (52). Evolutionary relationships under this new analysis differ slightly from our genome-scale phylogeny (Fig. S2 at https://doi.org/10.6084/m9.figshare.21382131). Additional genome sequences of species and strains may shed light on the evolutionary history of these species (54).

#### *Aspergillus flavus* and *Aspergillus oryzae*

Accurate classification of *A. flavus, A. oryzae*, and *Aspergillus parasiticus* is important because of biomedical and food safety concerns (Fig. 3f). *A. flavus* is a human pathogen, post-harvest food pathogen, and mycotoxin producer (19, 55, 56). *A. oryzae* is the domesticate of *A. flavus* used for food fermentation (e.g., sake and miso production) (20). Successive short branch lengths and non-monophyly of *A. flavus* and *A. oryzae* strains may suggest multiple domestication events or the introduction of domesticated isolates into the wild. Another interpretation is that the two species are distinct ecotypes, rather than distinct species or populations. Notably, *A. oryzae* strains are known to produce fewer mycotoxins than *A. flavus* (20), which may be a diagnostic signature (i.e., phenotype) of an ecotype appropriate for food production.

Interestingly, the genomes of NRRL 2999 and E136, which represent strains of the aflatoxigenic post-harvest pathogen *A. parasiticus*, reside in a clade with *A. flavus* and *A. oryzae* strains. Notably, the two *A. parasiticus* strains do not form a monophyletic group. Similar to a previous report, strain NRRL 2999 is misidentified as *A. parasiticus* but is, in fact, *A. flavus* (22). Five strains of *A. flavus* are sister to *A. minisclerotigenes* and more distantly related to other *A. flavus* strains; our genome-scale analyses are also consistent with inferences based on a recent examination of individual barcode loci (22) in suggesting that the five *A. flavus* isolates are misidentified. Reexamination of the taxonomic loci by conducting sequence similarity searches against a gold standard database among these five isolates supported this finding (Table S3 at https://doi.org/10.6084/m9.figshare.21382131).

#### Misidentified/mislabeled reference genomes

Surprisingly, we identified instances where the type strain of a species (i.e., the representative isolate for a species) was misidentified/mislabeled. For example, phylogenomics indicated *Aspergillus bombycis* NRRL 26010 is mislabeled and is, in fact, *Aspergillus luteovirescens* (57). This finding raises awareness about how formerly used taxonomic names persist in databases. A similar observation was made for five species from the section *Nigri* and one species from the series *Versicolores* (Table 1). Thus, the genomes of seven strains are mislabeled as type strains in NCBI, which may misguide various experiments.

#### Species determination for unlabeled genomes

We also identified 11 isolates with unknown species designations (Table 1 and S6 at https://doi.org/10.6084/m9.figshare.21382131). Phylogenomics confidently provided species labels for most isolates. For example, strain GbtcF2 was confidently determined to be *A. flavus* because phylogenomics revealed GbtcF2 is monophyletic with other *A. flavus* isolates (Fig. 3f). However, we identified instances where population-level genome sequences were unavailable, which limited insights from phylogenomic analyses. For example, although sequence similarity searches of taxonomically informative loci against a gold standard database indicated that strain ADI1 is *Aspergillus pseudodeflectus*, the lack of genome sequences from this species made it impossible to make this inference from our phylogenomic analyses.

Similarly, there is no phylogenomic-based guidance for identifying novel species. For example, phylogenomics was unable to determine the species designation of strain MEXU 27854; however, sequence similarity searches of taxonomically informative loci against other *Aspergillus* genomes indicated that strain MEXU 27854 is likely a novel *Aspergillus* species (Table S3 at https://doi.org/10.6084/m9.figshare.21382131).

#### Mislabeling among outgroup taxa

While confirming species designations using taxonomically informative loci, misidentified outgroup taxa were also identified. Specifically, *Penicillium chrysogenum* P2niaD18 and *Penicillium coprophilum* IBT 31321 are misidentified; instead, isolates P2niaD18 and IBT 31321 are *Penicillium rubens* and *Penicillium brevistipitatum*, respectively. Similarly, strain MCCF 102 is not an *Aspergillus* but a *P. formosus* (Table 1).

These findings suggest that issues of species, even genus, misidentification are prevalent both within and beyond *Aspergillus*, raising the possibility that misidentification is pervasive across microbes. Indeed, misidentification has been reported in bacterial genera such as *Burkholderia* and *Streptococcus* (58, 59) and genera of *Saccharomycotina* yeasts such as *Candida* and *Naumovozyma* (60, 61). Genome sequencing of multiple strains, including the type strains, of more *Aspergillus* species will further facilitate phylogenomic-based taxonomy.

### A roadmap for studies of *Aspergillus*

**Fig 4:**
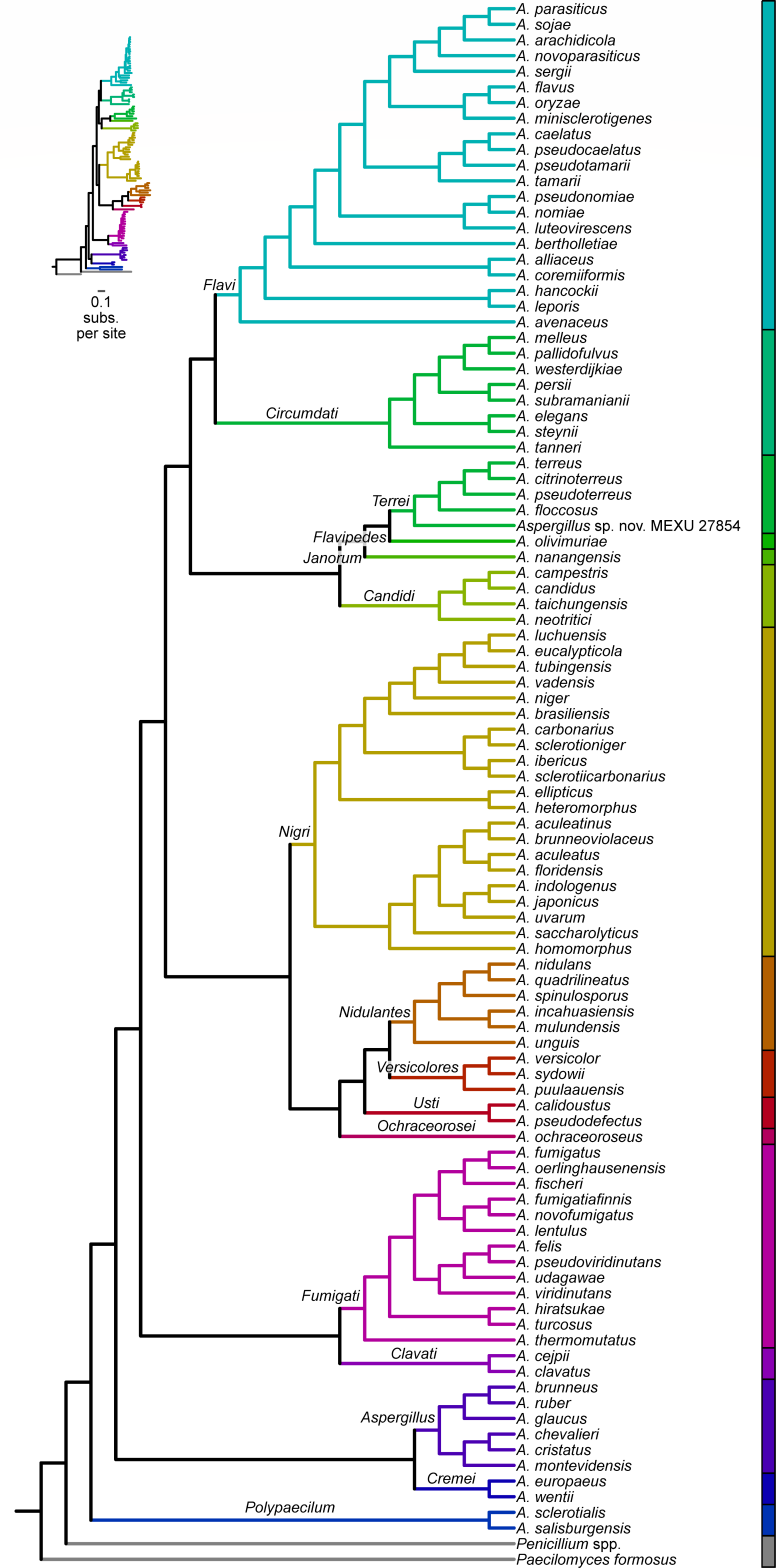
A species-level phylogeny of the genus *Aspergillus*. A genome-scale view of the *Aspergillus* phylogeny and the identification of a new sister lineage, clade A, to a clade of the rest of *Aspergillus* species whose genomes have been sequenced may help understand the early evolution of this biomedically and technologically relevant lineage. Inset represents the same phylogeny with branch lengths representing substitutions per site. Note, *Versicolores* is a series within the section *Nidulantes*.

We present a comprehensive genome-scale phylogeny of *Aspergillus* (Fig. 1 and 4). Our results underscore the need for further research into *Aspergillus* species delimitation and that strain misidentification may be a more common problem among publicly available data than previously appreciated (22). Strain misidentification can be reduced by two factors: genome-scale data, which are less prone to errors in phylogenetic inference compared to single or a few loci (23, 33, 38, 62–64), and population-level sampling from diverse environmental niches and geographic locations. Combined, these two factors facilitated identifying cases where strains may represent distinct species (such as *A. neoellipticus*) or were misidentified (such as the case of *A. niger* and *A. welwitschiae* and species in section *Flavi*). To facilitate others using our findings, we summarize higher-order and species relationships among *Aspergillus* species (Fig. 4).

Resolving these issues and facilitating future identification of strains and species requires prioritizing the genome sequencing of type strains. However, we identified that seven reference genomes were mislabeled in NCBI, suggesting that genome sequencing of multiple strains for each species will also be helpful. Genome-scale resources can be utilized for strain classification, similar to databases of internal transcribed spacer regions of fungi that help facilitate isolate and strain classification (6, 65–68). This approach is of growing interest among biologists working on *Saccharomycotina* yeasts and cryptic species of *Aspergillus* pathogens (23, 26, 28, 69, 70), but has yet to be well adapted for other fungal lineages. Currently, analysis of taxonomically informative loci remains the gold standard and should be cross-referenced with the latest, accepted taxonomic information available (54, 71). Working with NCBI curators to correct known mislabeling issues should also be a priority for members of the research community.

Adopting a genome-scale approach will require extensive genome sequencing efforts, such that publicly available genome sequence information is on par with publicly available single-locus information and well-curated genome-scale markers. To this end, we have produced profile Hidden Markov Models of the 1,362 molecular markers used in the present study that can facilitate genome-scale determination of *Aspergillus* species (see Data Availability). These markers, combined with our phylogenomic tree, may be helpful resources for accurate species determination among newly sequenced *Aspergillus* genomes. Coupling genome-scale data with phenotypic information may also help resolve certain controversies where DNA alone is insufficient. Incorporating phenotypic information will also help leverage copious amounts of data and taxonomic information generated prior to the genomics era (51, 72). Moreover, integrating phylogenomic and high-throughput phenotypic information may unite traditional taxonomic approaches with big data, allowing for multiple dimensions of information to elucidate species boundaries and determinations, an approach referred to, by us and others, as “taxogenomics” (73–76).

Additional research is needed to identify and characterize cryptic species, organisms morphologically highly similar to known species but genetically and physiologically distinct (23, 56, 77). Accurate strain identification and elucidation of species boundaries will greatly benefit from increased genome sequencing of under-represented species and populations thereof.

### Conclusion

Using a phylogenomic framework, dense sampling of genes, species, and populations is helpful for species identification. We demonstrate this using biomedically and technologically important fungi from the genus *Aspergillus* using 725 genomes and 1,362 high-quality molecular markers. In doing so, we resolved ongoing taxonomic controversies, identified new ones, and revealed extensive strain misidentification. Together, these analyses underscored the considerable amounts of inaccurate information concerning *Aspergillus* genomes and the importance of population-level and species-level sampling for identifying these inaccuracies. Our finding that some outgroup taxa were misidentified and that one isolate labeled as *Aspergillus* was *Paecilomyces* indicates that the issue of strain misidentification may be pervasive among microbes. Our ability to identify and resolve taxonomic controversies was aided by a multi-disciplinary approach, leaning on expertise from mycologists, taxonomists, bioinformaticians, and phylogenomicists. Although our approach was efficacious, it relies on the availability of genome sequences from type strains and populations. Thus, we advocate for additional genome sequencing across the genus and suggest that our approach is best supported by the traditional identification method. However, as the number of genomes increases across the Tree of Life rapidly, we anticipate that our framework and collaborative approach can be broadly applied to ensure accurate species identification.

## MATERIALS AND METHODS

### Genome data acquisition and quality control

All publicly available *Aspergillus* genomes (*N* = 717) were downloaded from NCBI (National Center for Biotechnology Information; https://www.ncbi.nlm.nih.gov/; date accessed: 9 January 2022). Publicly available genome annotations were also downloaded. For genomes without available annotations, gene boundaries were predicted using AUGUSTUS, v3.3.2 (78), with the “species” parameter set to “aspergillus_nidulans.” To determine if the genomes were suitable for phylogenomic analyses, gene prediction completeness was examined using BUSCO, v4.0.4 (79), and the Eurotiales database of 4,191 near-universally single-copy orthologs (or BUSCO genes) from OrthoDB, v10 (80). Six genomes with less than 75% single-copy complete BUSCO genes were removed, resulting in 711 genomes labeled as *Aspergillus* in NCBI. The resulting sets of gene predictions were highly complete (mean ± standard deviation: 95.74% ± 2.25%). For outgroup taxa, 14 *Penicillium* genomes and annotations were downloaded from NCBI. The completeness of *Penicillium* gene predictions was assessed using the same protocol and was highly complete (mean ± standard deviation: 94.73% ± 4.04%). The final data set had 725 genomes.

### Single-copy orthologous gene identification

Phylogenomics often relies on single-copy orthologous genes. OrthoFinder, v.2.3.8 (81), was used to identify single-copy orthologous genes by clustering protein sequences into groups of orthologs. Clustering sequences were based on protein sequence similarity and calculated using DIAMOND, v2.0.13.151 (82). To reduce computation time and memory, orthology predictions were conducted among 40 representative species that span the diversity of *Aspergillus* species (Table S7 at https://doi.org/10.6084/m9.figshare.21382131) (33). The impact of 41 different inflation parameter settings (one through five with a step of 0.1) on the number of single-copy orthologs identified was examined. The inflation parameter that resulted in the highest number of single-copy orthologs was 3.6. The resulting 7,882 single-copy orthologs with at least 50% occupancy (*N* = 20) were used for downstream analysis.

To identify orthologs in the full 725-genome data set, sequence similarity searches were conducted in each proteome. To do so, the 7,882 single-copy orthologs were aligned using MAFFT, v7.402 (83), with the auto parameter. Profile Hidden Markov Models (HMMs) were then built for each alignment using the hmmbuild function in HMMER, v3.1b2 (84). The resulting HMMs were used to identify single-copy orthologs in the 725 proteomes using orthofisher, v.1.0.3 (85), and a bitscore fraction threshold of 0.95.

To generate single-gene phylogenies, the protein sequences of the 7,882 single-copy orthologs, identified using orthofisher (85), were aligned using MAFFT as described above (83). The corresponding nucleotide sequences were threaded onto the protein alignment using the thread_dna function in PhyKIT, v1.11.12 (86) and trimmed using ClipKIT, v1.3.0 (87). Excessive trimming of multiple sequence alignments worsens single-gene phylogenetic inference (87, 88); thus, multiple sequence alignments wherein 40% or more of the original alignment length was maintained after trimming were retained resulting in 4,300 single-copy orthologs. The evolutionary histories of the 4,300 single-copy orthologs were inferred using IQ-TREE 2 (89). The best-fitting substitution model was selected using ModelFinder (90). To remove potential instances of hidden paralogy, the monophyly of the five well-established lineages was examined using PhyKIT (86). Specifically, the single-gene phylogenies were examined for the monophyly of five well-established lineages: sections *Flavi* (*N* = 246), *Fumigati* (*N* = 316), *Nidulantes* (*N* = 12), and *Versicolores* (*N* = 7) as well as the outgroup lineage of 14 *Penicillium* species. Genes, wherein one or more of the five lineages were not monophyletic, were removed, resulting in a final set of 1,362 single-copy orthologous genes. The average occupancy for each single-copy ortholog was 0.98 ± 0.07.

### Phylogenomic tree inference

The final set of single-copy orthologs was used to infer the evolutionary history of the 725 species and strains. Specifically, the 1,362 single-copy orthologs were concatenated into a single matrix using the create_concat function in PhyKIT (86). The resulting supermatrix had 6,378,237 sites (2,846,432 parsimony informative sites). The alignment length and number of parsimony informative sites were calculated using BioKIT, v0.1.2 (91). IQ-TREE 2 was used for tree inference (89). The best-fitting substitution model was selected using ModelFinder (90). Bipartition support was assessed using 1,000 ultrafast bootstrap approximations (92). The total central processing unit (or CPU) hours required for this analysis was 2,763 (approximately 4 months). An additional coalescence-based consensus tree approach was used to infer the evolutionary history among species and strains. Specifically, the 1,362 single-copy orthologous gene phylogenies were input to ASTRAL, v5.6.3 (93). Phylogenies were visualized using iTOL, v6 (94).

### Species confirmation using taxonomic loci

The current standard for species identification is sequence similarity using taxonomically informative loci. To conduct species determination using taxonomically informative loci, the best nucleotide-to-nucleotide BLAST hit to beta-tubulin, calmodulin, and/or RNA polymerase II second largest subunit (RPB2) gene regions were extracted from the genome assembly of each isolate. The query sequence for each sequence is as follows: calmodulin, GenBank identifier: EF669865.1, Description: *Neosartorya fischeri* isolate NRRL 181 calmodulin gene, partial cds; beta-tubulin, GenBank identifier: EF669796.1, Description: *Neosartorya fischeri* isolate NRRL 181 beta-tubulin gene, partial cds; and RPB2, GenBank identifier: XM_677297.2, Description: *Aspergillus nidulans* FGSC A4 DNA-directed RNA polymerase II core subunit RPB2 (ANIA_09120), partial mRNA. A sequence similarity search was conducted for each extracted sequence against a local database of taxonomically informative fungal sequences. The database comprised reference sequences sourced from GenBank, encompassing the accepted *Aspergillus* species listed by Houbraken et al. (54), as well as the new *Aspergillus* species described afterward.

## Data Availability

Results and supplementary data presented in this study are available from figshare at https://doi.org/10.6084/m9.figshare.21382131.
